# Meeting Report: Application of Genotyping Methods to Assess Risks from *Cryptosporidium* in Watersheds

**DOI:** 10.1289/ehp.8240

**Published:** 2005-10-26

**Authors:** Christobel Ferguson, Dan Deere, Martha Sinclair, Rachel M. Chalmers, Kristin Elwin, Stephen Hadfield, Lihua Xiao, Una Ryan, Robin Gasser, Youssef Abs El-Osta, Melita Stevens

**Affiliations:** 1 Ecowise Environmental, Canberra, Australia; 2 Cooperative Research Centre for Water Quality and Treatment, Adelaide, Australia; 3 University of New South Wales, Sydney, Australia; 4 Sydney Catchment Authority, Sydney, Australia; 5 Department of Epidemiology and Preventive Medicine, Monash University, Melbourne, Australia; 6 National Public Health Service for England and Wales, Swansea, United Kingdom; 7 Centers for Disease Control and Prevention, Atlanta, Georgia, USA; 8 Murdoch University, Perth, Australia; 9 Melbourne University, Melbourne, Australia; 10 Melbourne Water, Melbourne, Australia

**Keywords:** *Cryptosporidium*, drinking water, genotyping, watersheds

## Abstract

A workshop titled “Application of Genotyping Methods to Assess Pathogen Risks from *Cryptosporidium* in Drinking Water Catchments” was held at the International Water Association biennial conference, Marrakech, Morocco, 23 September 2004. The workshop presented and discussed the findings of an interlaboratory trial that compared methods for genotyping *Cryptosporidium* oocysts isolated from feces. The primary goal of the trial and workshop was to assess the utility of current *Cryptosporidium* genotyping methods for determining the public health significance of oocysts isolated from feces in potable-water–supply watersheds. An expert panel of 16 watershed managers, public health practitioners, and molecular parasitologists was assembled for the workshop. A subordinate goal of the workshop was to educate watershed management and public health practitioners. An open invitation was extended to all conference delegates to attend the workshop, which drew approximately 50 interested delegates. In this report we summarize the peer consensus emerging from the workshop. Recommendations on the use of current methods by watershed managers and public health practitioners were proposed. Importantly, all the methods that were reported in the trial were mutually supporting and found to be valuable and worthy of further utility and development. Where there were choices as to which method to apply, the small-subunit ribosomal RNA gene was considered to be the optimum genetic locus to target. The single-strand conformational polymorphism method was considered potentially the most valuable for discriminating to the subtype level and where a large number of samples were to be analyzed. A research agenda for protozoan geneticists was proposed to improve the utility of methods into the future. Standardization of methods and nomenclature was promoted.

Of the > 150 potentially waterborne pathogens [World Health Organization (WHO) 2004], *Cryptosporidium* is the most notorious in developed countries, having been the suspected etiologic agent of large waterborne disease outbreaks (Rose et al. 1997; Thompson et al. 2003). The most striking evidence of the significance of *Cryptosporidium* to water authorities is that regulations and guidelines have been introduced specifically to deal with it. For example, in response to outbreaks of cryptosporidiosis in 1995 and 1997, new regulations were introduced in England and Wales, which required testing and reporting against legally binding targets for *Cryptosporidium* concentrations that could be found in treated drinking water [Drinking Water Inspectorate (DWI) 1999]. In the United States, a series of regulations under the Safe Drinking Water Act (U.S. Environmental Protection Agency 1998) required the monitoring and removal of *Cryptosporidium* in response to a series of waterborne cryptosporidiosis outbreaks, including the largest ever recorded, with an estimated 400,000 cases [MacKenzie et al. 1994; possibly overestimated through self-reporting bias (Hunter and Syed 2001)]. In Sydney, Australia, a series of boil water advisories in 1998 followed high-level *Cryptosporidium* detections in the city’s water supply (Cox et al. 2003). Although no cases of illness were associated with the incident, the Australian water industry responded by voluntarily adopting externally audited risk management systems based on hazard analysis and critical control points (HACCP) (Deere and Davison 1998; Havelaar 1994). In comparison, England and Wales have adopted a regulatory approach requiring water utilities to monitor *Cryptosporidium* in raw and finished drinking waters (DWI 1999; McCann 1999).

## Risk-based water-quality management.

Since first promoted by Stevens et al. (1995), the value of adopting a risk-based approach to water-quality management, for both public health and aesthetic aspects, has prevailed over the adoption of increased emphasis on end point testing-based approaches (Davison et al. 2005; WHO 2004). Risk-based water-quality management principles can be applied to watershed management (Barry et al. 1998; Deere 2004). Importantly, the adoption of a risk-based approach requires a sound scientific evidence base (termed validation) for the statements made in relation to the safety of water (Davison et al. 2005; WHO 2004). Because of intense focus on *Cryptosporidium* in water safety management, the validation of drinking-water risk management plans requires particular attention to the assumptions relating to this pathogen.

## The importance of genotyping Cryptosporidium oocysts.

*Cryptosporidium* oocysts are detected at appreciable concentrations, often 10^3^/g and sometimes reaching > 10^7^/g, in the feces of a wide variety of the wild and domestic animals (including humans) found in watersheds (Cox et al. 2005; Fayer et al. 2000; Ryan et al. 2005). However, there is evidence that the public health significance of most of these oocysts is limited because of a broad genetic diversity within the *Cryptosporidium* genus (Xiao et al. 2004). Human sewage and manure from domestic cattle and sheep are probably the most important sources of human-infectious *Cryptosporidium* (Thompson et al. 2003), with most cryptosporidiosis cases predominantly being caused by just two (*C. hominis* and *C. parvum*) of the 15 named *Cryptosporidium* species (Xiao et al. 2004) and just a fraction of the > 30 described genotypes. However, routine microscopic detection methods do not discriminate among species, genotypes, or subtypes (Thomas and Chalmers 2003), leaving water authorities with data indicating *Cryptosporidium* presence but no indication of the health significance of this finding.

Several benefits arise from better understanding the public health significance of *Cryptosporidium* oocysts in watersheds, including significant financial implications. First, interventions within the watershed can be better targeted to the most important land-based sources. Second, interventions downstream of the watershed can be proportional to the risk. For example, New York (USA) and Melbourne (Australia) do not provide a water treatment intervention to remove or inactivate *Cryptosporidium* oocysts from their better protected watersheds, relying instead on watershed protection and large storage reservoirs. However, these and many similar towns and cities face continued pressure to spend hundreds of millions of dollars on implementing additional water treatment for *Cryptosporidium*. In other cases, boil water advisories have been issued when oocyst numbers from watersheds reached levels of concern. Therefore, understanding the public health significance of oocysts circulating within a specific watershed, its subwatersheds, and the associated fauna would enable decisions relating to all three areas—watershed management, water treatment, and emergency response—to be better targeted and public money to be most cost effectively appropriated.

There are a number of means of objectively assessing the public health significance of any particular isolate of *Cryptosporidium* oocysts. The most unequivocal approach is to feed oocysts to human volunteers (Chappell et al. 1999; DuPont et al. 1995; Messner et al. 2001; Okhuysen et al. 1999). However, with 15 accepted species and > 30 genotypes now described, this approach is considered too costly and time-consuming to be practically applied to more than a fraction of that number as well as having ethical and safety considerations. Furthermore, experimental infection is not necessarily a good indicator for natural infectivity, with some animal feeding trials failing to show infection in animals with parasite isolates from the same species—for example, *C. andersoni* in cattle. Epidemiologic analysis of the safety of a whole water supply system is feasible at reasonable cost (Hellard et al. 2001). However, most of the risk of *Cryptosporidium* exposures from water supplies would arise under extreme event scenarios when, for example, a large storm might coincide with a high level of human-infectious *Cryptosporidium* genotype prevalence in a watershed and a treatment plant failure (Davison et al. 1999; Stevens et al. 1995). Therefore, earlier warning than that provided by epidemiologic studies is desired by water and health authorities. The approach considered in this report is to quantify and type oocyst sources in watersheds and compare those with isolates from human patients. The results permit hypotheses to be tested regarding the similarity of genotypes propagating within watershed hosts and those propagating within human populations consuming the water abstracted from those watersheds.

Molecular genetic methods of discriminating pathogens to support epidemiologic investigations can be applied to help assess the public health significance of *Cryptosporidium* oocysts in watersheds (Thompson 2000). There are some difficulties in applying such techniques to the small numbers of degraded oocysts, of mixed population, that are isolated from water samples. Therefore, the focus of this report and the workshop described is the analysis of fresh material from human or animal feces.

## Workshop Objectives

An international trial of methodologies and a workshop were undertaken to compare currently available methods for genotyping *Cryptosporidium* for assessing the health significance of oocysts isolated from fecal stool samples from watersheds. Full technical details of the trial and the methodologies used are given in a separate report (Chalmers et al. 2005). The workshop, titled “Application of Genotyping Methods to Assess Pathogen Risks from *Cryptosporidium* in Drinking Water Catchments,” was held as part of the International Water Association Biennial Conference in Marrakech, Morocco, on 23 September 2004. Before the workshop, an international trial was held in five laboratories across three continents to provide an objective first assessment of some currently available methods of *Cryptosporidium* genotyping and subtyping in terms of their discriminative ability, ease of use, practicality, robustness, and repeatability. The isolates used for the interlaboratory trial were from fecal samples and were selected on the basis of available case data to enable epidemiologic information to be considered when interpreting results. The primary goal of the workshop was to gain an international peer consensus on the utility of these current *Cryptosporidium* genotyping methods for determining the public health significance of oocysts isolated from feces in watersheds. Additional goals of the workshop included developing a research agenda, a consensus on standardization of both nomenclature and methodologies, and the technology transfer of methods. A total of 16 invited participants contributed (Appendix 1) to the workshop, and > 50 delegates were in attendance, many of whom provided very valuable commentary.

## Discussion and Workshop Results

### Comparison of methods tested.

Unpassaged *Cryptosporidium* oocysts from a total of 42 human, five lamb, and three calf rectal swab or stool samples were selected from six epidemiologically and two nonepidemiologically based collections and DNA extracts were prepared from each (Table 1). The genotyping methods considered at the workshop and during the interlaboratory trial are summarized in Table 2, which includes a simplistic analysis of their relative utility.

Some limitations were common to all the methods tested in relation to typing oocysts recovered from water samples and are not identified in Table 2. Watershed managers can send *Cryptosporidium*-positive water samples to analytical laboratories for genotyping and subtyping (Jiang et al. 2005; Ryan et al. 2005; Xiao et al. 2000, 2001; Zhou et al. 2003). Such results have proved valuable to water authorities, preventing unnecessary alarm where *Cryptosporidium* oocysts in water sampled from protected watersheds were shown not to be human pathogenic genotypes (Jiang et al. 2005; Xiao et al. 2000; 2001). Oocyst populations isolated from water are often of mixed genotype (Jiang et al. 2005; Xiao et al. 2000), very low (< 10) in number [necessitating nested polymerase chain reaction (PCR) with its higher risk of artifacts], and may have lost their nucleic acid content, which is in any case likely to coextract with PCR inhibitors. Furthermore, all the methods considered used nuclear DNA as the target for discrimination, and thus the methods do not necessarily discriminate viable oocysts from those that are nonviable but are otherwise intact. Finally, it is difficult and costly to set up the typing methods in a form where they provide accurate quantitation of oocyst numbers present (quantitative PCR or serial dilution and most probable number methods). Therefore, in general, conventional immunofluorescent antibody oocyst enumeration would need to be undertaken in support of typing if quantitative data are required. Despite these limitations, there was strong agreement from watershed managers and public health practitioners that the methods were a valuable and important component of characterizing the health-risk implications of *Cryptosporidium* oocysts present in the feces of animals in watersheds.

Although it was possible to simplistically rank the methods based on their relative performance characteristics (Table 2), this was considered an overly simplistic approach, and there was consensus that all the methods described performed very well and yielded valuable results. Furthermore, the inferences made from the results of the various methods were not contradictory. The precise choice of which method might be the best in any particular circumstance would depend on the question being asked by the practitioner as well as practical considerations, such the capability and experience of local laboratories.

### Utility of methods.

It was noted that, at the detailed level, the methods would evolve and improve over time. Therefore, it was agreed that a first point of consensus needed to be on the preferred genetic locus to target because this need not change over time. Agreement now on the optimum or consensus locus would help future researchers compare future with past results. In the context of *Cryptosporidium*, genotyping refers to differentiation between different species and genotypes and requires conservative loci such as the small-subunit ribosomal DNA (*SSU rDNA* or *SSU rRNA*) (GenBank accession no. L16996; all accession numbers from the GenBank Database, http://www.psc.edu/general/software/packages/genbank/genbank.html) and the 70-kDa heat-shock protein (*HSP70*) locus (GenBank accession no. AF221528). Subtyping involves looking at variation within a particular species or genotype and requires much more variable loci such as the hyper-variable 60-kDa glycoprotein (*GP60*) gene (GenBank accession no. AF164489) and microsatellites.

The genotyping methods currently used differ with respect to the genetic locus targeted: the *SSU rRNA* gene, the *HSP70* gene, or the *GP60* gene. All are acceptable, although there was consensus that the *SSU rRNA* gene (*SSU rDNA*) was the optimum locus for genotyping for several reasons. The *SSU rDNA* locus is repetitive with a copy number of five, which improves method sensitivity. The locus has been the most commonly targeted for phylogenetic analyses and has the largest historical database worldwide. There are genus-specific primers that amplify the *SSU rDNA* gene for all 15 species and the > 30 genotypes thus far described, which is of particular relevance to environmental studies that include wildlife.

For subtyping, the second internal transcribed spacer region of nuclear ribosomal DNA (*ITS-2*) (GenBank accession no. AF015774) was considered the optimum locus and is thought to be sufficiently variable as well as being present in multiple copy number (five).

A locus not targeted in any of the methods tested, but one that should not be overlooked and is of value is the *Cryptosporidium* oocyst wall protein (*COWP*) gene (GenBank accession no. AF266273), which is useful when there is ample material from which to extract DNA, a large number of samples are to be screened, and *C. parvum* or *C. hominis* species are likely to dominate, such as for clinical isolates.

The relative value of going to the genotyping or subtyping level was considered. There was not a consensus view from the workshop participants, although it was noted that genotyping as a first pass might be adequate in many studies provided there was sufficient DNA remaining after the genotyping to perform subtyping where required.

In application, there was consensus that *Cryptosporidium* typing methods could be used in both a reactive and proactive sense. Reactive analyses would involve responding to cryptosporidiosis outbreaks or *Cryptosporidium* oocyst contamination incidents by typing oocysts from cases and/or contaminated water samples and comparing results with those from putative watershed sources. However, there was concern that such applications are both untimely in terms of public health protection and potentially misleading. False-positive conclusions could be supported because commonality of genotype does not equate to cause and effect. False negative conclusions could be supported because, by definition, sampling captures only a fraction of the whole and may miss the true watershed source. Despite these limitations, reactive typing was considered worthwhile to assist epidemiologists in testing hypotheses relating to associations between water exposure and cryptosporidiosis cases.

A systematic and proactive analysis was endorsed as the higher priority for watershed managers and public health practitioners by the workshop participants. The typing methods would be used to type oocysts isolated from putative watershed sources and compare results with oocysts from sporadic human cases of disease. If possible, an ongoing study should be performed rather than an ad hoc snapshot. Over time, an understanding of the epizoology and epidemiology of oocysts in the human and animal populations of the watershed would build up, which could be related to similar data for those consuming the yielded water and other typing databases from human cases. The resulting database could be used in testing hypotheses relating to the public health significance of oocysts in the watershed. However, typing studies were understood not to be simple or short-term projects. The temporal and spatial variability in *Cryptosporidium* types within any particular watershed, as well as the presence of non-*C. parvum* and non-*C. hominis* infections in some clinical cases, imply the need for great care in both the design and interpretation of water-related *Cryptosporidium*-typing projects.

### Research priorities.

At the practical level, the participants agreed that improved standardization in nomenclature combined with more consistent methodologies would greatly facilitate the comparison of isolates from different geographic areas and help to better establish the infectivity or virulence of subtypes. The value of technology transfer between laboratories and other forms of enhanced collaboration between the participants to the trial and other genetic researchers was endorsed. The nomenclature applied to *Cryptosporidium* phylogeny was rapidly evolving, and it was acknowledged that this is an area that may remain imperfect for the foreseeable future. However, continued efforts to improve consistency were considered worthwhile. An example of this problem cited at the workshop was that one of the trial isolates was typed as three different genotype names in GenBank—cervid, mouflon sheep, and lemur—depending on the reporting laboratory.

Further characterization of the *ITS-2* locus for subtyping was considered necessary. There was an as yet unrejected hypothesis that some of the variation seen was due to amplification of the *SSU rDNA* B-type subunit gene. Of the five copies of the *SSU rDNA* of *C. parvum*, four copies code for type A and one for type B (this ratio can differ for other species). There are genetic differences between types A and B in the regions incorporated into ribosomes as well as an approximately 9% difference within the same isolate in the *ITS-2* region (Ryan U, unpublished observations). Therefore, a limitation of the *ITS-2* locus is that some intraisolate variation could be encountered after amplification of the B subtype.

Single-strand conformation polymorphism (SSCP) is a gel-based tool that can be applied to any locus and was further considered because of its performance (Chalmers et al. 2005). Further work will evaluate whether all currently described genotypes can be identified and differentiated based on the SSCP analysis of the *SSU rDNA* and/or *ITS-2* loci and will assess interlaboratory reproducibility of the method.

There are a number of limitations with reliably and routinely analyzing environmental samples, and two areas of research are required to overcome these. Methodologic preparative techniques need to be improved to help capture more oocysts and recover more of their DNA. In addition, the ability of genotyping methods to discriminate and identify the genotypes and subtypes present in mixed populations of oocysts needs to be improved. The former area of research was considered a higher priority because, if required, dilution can be used to reduce DNA concentrations to the point where only one genotype or subtype is present in any aliquot.

Limited studies have found genotype and subtype sequences to be stable on passage although more analysis of stability is warranted. However, because the purpose of the genotyping is to assess public health significance, more fundamental research that develops an understanding of the human-specific virulence factors and their relationship to genotype is a higher priority. It was noted that, for the loci analyzed thus far, virulence can vary significantly for the same genotype or subtype, and there may turn out to be a poorer than desired relationship. For example, the first three oocyst isolates used in human feeding trials were of the same *GP60* subtype but had differences of two orders of magnitude in infectivity as well as leading to different symptom severities. Another concern was raised relating to biases that arise because of *Cryptosporidium* subtypes that lead to the greatest symptom severity predominating in the human-case databases by reporting bias. However, this was agreed to be a self-selecting bias that actually helped in setting public health priorities for watershed management because the subtypes that cause the most severe symptoms are of most publication health concern. It was further noted that the use of cell lines for infectivity assessment had been shown to be less than perfectly correlated with infectivity in humans and certainly not related to symptom severity after infectivity. For example, *Cryptosporidium andersoni* can infect commonly used HCT-8 cell lines but does not appear to be infectious and pathogenic in humans.

## Conclusion

All the methods reported were consistent with one another and were agreed by the workshop participants to be acceptable and capable of genotyping *Cryptosporidium* oocysts from animal and human fecal samples. If choices were to be made as to which method to apply in any particular circumstance, the *SSU rDNA* was considered the optimum locus to target for reasons that included the size of the current database and the availability of primers that would detect the presence of all known genotypes. Partly because of the ready availability of sequencers, the sequencing-based methods were considered the most generally applicable if discrimination was only required to the genotype level. However, where a large number of samples were to be analyzed, the SSCP method had a possible advantage because of its lower cost and may become the default genotyping method where a large number of samples were to be analyzed. SSCP was also considered the most promising method for discriminating at the subtype level. However, specific hypotheses relating to a number of the methods, including SSCP, need to be tested, including questions regarding their ability to detect all genotypes of *Cryptosporidium* and uncertainties relating to the true source of subtype variation. Standardization of the typing methods and of the nomenclature was promoted to increase the value of the results for watershed managers and public health practitioners.

## Figures and Tables

**Appendix 1:** Invited workshop contributors.

Christobel Ferguson	Sydney Catchment Authority and CRCWQT, Sydney, Australia
Keith Osborn	United Utilities, Warrington, U.K.
Daniel Deere	Water Futures and CRCWQT, Sydney, Australia
Kristin Elwin	U.K. Cryptosporidium Reference Unit, NPHS, Swansea, U.K.
Hans Albrechtsen	Technical University of Denmark, Lyngby, Denmark
Leo Heijnen	KIWA, Utrecht, Netherlands
Jonathon Wastling	SPDL, Glasgow, U.K.
Lihua Xiao	CDC, Atlanta, GA, USA
Marilyn Marshall	University of Arizona, Tucson, AZ, USA
Ramon Aboytes	American Water, Belleville, WA, USA
Melita Stevens	Melbourne Water and CRCWQT, Melbourne, Australia
Stephen Hadfield	U.K. Cryptosporidium Reference Unit, NPHS, Swansea, U.K.
Nick Ashbolt	University of NSW and CRCWQT, Sydney, Australia
Una Ryan	Murdoch University, Perth, Australia
Rachel Chalmers	U.K. Cryptosporidium Reference Unit, NPHS, Swansea, U.K.
Youssef Abs El-Osta	Melbourne University, Melbourne, Australia

Abbreviations: CDC, Centers for Disease Control and Prevention; KIWA, Kiwa Water Research; NPHS, National Public Health Service; NSW, New South Wales; SPDL, Scottish Parasite Diagnostic Laboratory.

**Table 1:** Sources of oocysts used for the interlaboratory trial.

Source of feces	Location	Period	Epidemiology	Notes
22 human cases	Adelaide, Australia	2000 and 2001	Sporadic: case control study (Robertson et al. 2002)	3 immediate family pairs
6 human cases and 3 bottle-fed lambs	Wales, UK	2003	Outbreak: open farm (CDSC outbreak number 03/197)	
2 human cases and 1 scouring lamb, 1 asymptomatic lamb and 1 from calf barn effluent	Eastern England, UK	2003	Outbreak open farm (John Bailey and Susanna Williamson, personal communication)	
1 human and 2 calves	Northeastern England, UK	2002	Sporadic: private farm (Paul Duff, personal communication)	Human had access to calf pen
4 human cases	Wales, UK	2002	Outbreak: rural area (Debra Halstead, personal communication)	Private water supply and farm visits possible sources
4 human cases	Northeastern England, UK	2000	Outbreak: swimming pool (CDSC outbreak reference number 00/406)	
5 human cases	UK	Not stated	Sporadic	Each of these samples were supplied in duplicate to provide a single-blinded test for assay repeatability
2 human cases	UK	2002	Sporadic	1 sample was prepared as a 1:1 mixture for both to provide a single-blinded test for assay capability to discriminate mixtures

CDSC, Communicable Disease Surveillance Center.

**Table 2:** Multiple criteria analysis comparing the methods considered during the interlaboratory trial and discussion points from the International Water Association workshop.

Method	Turnaround [days (rank)]	Repeatability [duplicates (rank)]	Discrimination [no. of types (rank)]	Typeability [index (rank)]	Detected mixtures [in trial (rank)]	Overall rank
SSCP of nontranscribed *ITS*-region of *SSU rDNA*	1 (1)	5/5 (1)	8 (1)	0.96 (2)	Yes (1)	1
SSCP of transcribed region of *SSU rDNA*	1 (1)	5/5 (1)	3 (5)	0.93 (3)	Yes (1)	2
Sequencing of *GP60* DNA	2 (2)	4/5 (2)	7 (2)	0.78 (6)	Partial (2)	3
PCR-RFLP of *SSU rDNA*	4 (3)	5/5 (1)	5 (4)	0.98 (1)	Partial (2)	3
Multilocus genotyping at 3 microsatellites	2 (2)	2/5 (3)	6 (3)	0.90 (5)	Partial (2)	4
Sequencing of *HSP70*	4 (3)	5/5 (1)	3 (5)	0.92 (4)	No (3)	5

RFLP, restriction fragment length polymorphism.
